# Towards developing an organotypic model for the preclinical study and manipulation of human hair matrix-dermal papilla interactions

**DOI:** 10.1007/s00403-020-02178-8

**Published:** 2021-01-12

**Authors:** Christopher I. Platt, Jeremy Chéret, Ralf Paus

**Affiliations:** 1 Division of Cell Matrix Biology and Regenerative Medicine, The University of Manchester, Manchester, UK; 2 Dr. Phillip Frost Department of Dermatology and Cutaneous Surgery, University of Miami Miller School of Medicine, Miami, FL 33136 USA; 3 Monasterium Laboratory, Münster, Germany; 4 Centre for Dermatology Research, University of Manchester and NIHR Manchester Biomedical Research Centre, Manchester, UK

**Keywords:** Hair follicle, Spheroids, Cellular, Dermal papilla, Keratins, Hair specific, Cell communication, Drug development

## Abstract

Organ culture of microdissected scalp hair follicles (HFs) has become the gold standard for human ex vivo hair research; however, availability is becoming very limited. Although various simplistic “HF-equivalent” in vitro models have been developed to overcome this limitation, they often fail to sufficiently mimic the complex cell–cell and cell–matrix interactions between epithelial and mesenchymal cell populations that underlie the specific growth processes occurring in a native HF. Here, we have attempted to overcome these limitations by developing a novel human hair research model that combines dermal papilla (DP) fibroblasts, cultured as 3-dimensional (3D) spheroids (DPS), with plucked anagen hair shafts (HS). We show that DPS express HF inductivity markers, such as alkaline phosphatase (ALP), versican and noggin, while plucked HSs retain substantial remnants of the anagen hair matrix. When cultured together, DPS adhere to and surround the plucked HS (HS-DPS), and significantly enhance HS expression of the differentiation marker keratin-85 (K85; *p* < 0.0001), while simultaneously decreasing the percentage of TUNEL + cells in the proximal HS (*p* = 0.0508). This simple model may offer a physiologically relevant first step toward evaluating HF differentiation in the human anagen hair matrix.

**Supplementary Information:**

The online version contains supplementary material available at 10.1007/s00403-020-02178-8.

## Introduction

Current ex vivo and in vitro assays for human HF research include the HF organ culture model [16, 21], 3D organotypic models [1, 7, 9] and 2-dimensional (2D) co-culture of outer root sheath keratinocytes (ORSK) and DP fibroblasts [3]. Human HF culture is a highly relevant system for evaluating hair growth, and, using pharmacological and gene knockdown approaches, specific pathways regulating hair growth can be interrogated ex vivo [5, 10, 23]. However, surplus human scalp HFs from cosmetic procedures are becoming increasingly difficult to obtain, as more advanced surgical techniques have reduced the amount of skin available for research. This growing limitation demands the development of alternative models, which are also amenable for use in pre-clinical drug discovery [16]. This methodological study attempts to do that by combining cultured HF mesenchyme with plucked anagen scalp hair shafts (HS) in vitro.

As a minimum, in vitro hair models should contain cell types with key biological roles within the HF: follicular epithelial cells of the matrix, epithelial stem cells, mesenchymal cells of the DP and connective tissue sheath (CTS), melanocytes and immunocyte populations [13]. At its most basic, an in vitro model may attempt to recreate the signalling events between 2 distinct cell types in co-culture, such as inductive DP fibroblasts and ORSK [3]. Monolayer co-cultures are simple to achieve, inexpensive and can be scaled up for high-throughput analysis [19]. However, 2D culture does not reflect the contextual behaviour of native DP cells, as DP cells cultured in monolayer rapidly lose their inductivity [12]. In contrast, 3D culture of DP fibroblasts results in DP cells that are phenotypically and functionally akin to native DP [12]. In addition, 3D culture of ORSK in the presence of DP cells and basement membrane (matrigel) has been shown to induce ORS-like epithelial differentiation [9].

Here, we report the initial stages in the development of a novel in vitro model that balances the advantages of HF organ culture with the flexibility of 3D cell-based organotypic models. This model combines plucked human anagen hair shafts (HS) containing vital HF epithelium, with DP fibroblasts pre-cultured as 3D spheroids. Both epithelial and mesenchymal components of this model are derived from material that is easily obtained in relatively large quantity (plucked anagen HSs) and can be stored and/or expanded in the lab when required (DP spheroids).

## Materials and methods

### Skin samples

**Fig. 1:**
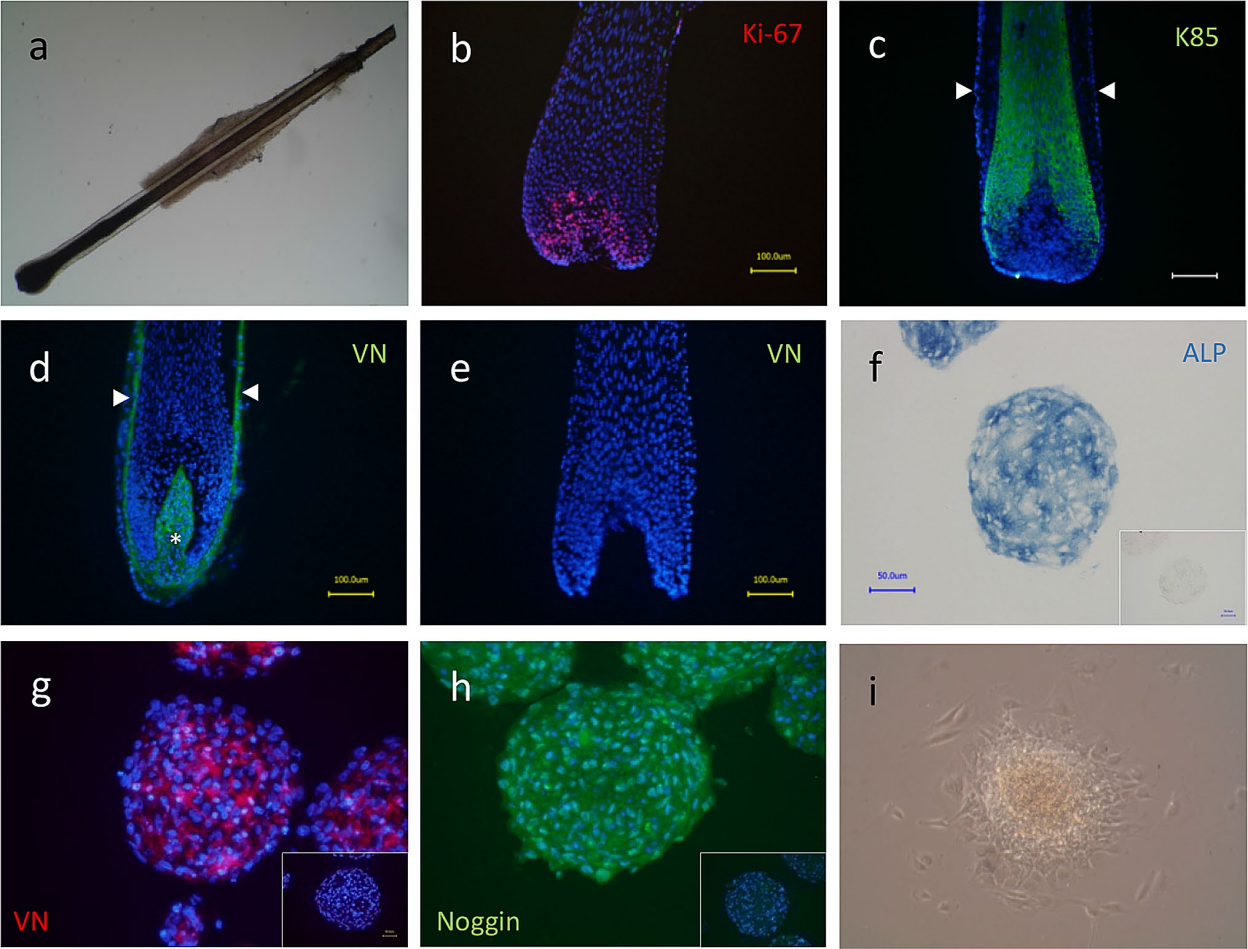
Light microscope image of a plucked scalp HS (**a**). Plucked HS showing Ki67 + cells (**b**), plucked HS showing anti-K85 IR (**c**), white arrow heads denote outer root sheath (ORS). Localisation of anti-versican IR in DP (asterisk) and CTS (white arrow heads) of intact anagen scalp HF (**d**), plucked HS stained with anti-versican (VN) showing absence of DP and CTS (**e**). ALP activity in DPS (**f**, inset shows DPS treated with ALP inhibitor, levamisole), anti-VN IR in DPS (**g**), anti-noggin IR in DPS (**h**), viability of DP fibroblasts 48 h after reseeding DPS on plastic substrate (**i**). Images of plucked HS are representative of three independent donors. Images of DPS are representative of cell cultures derived from three independent donors. Inset panels in **g** and **h** show no primary antibody control

This study was approved by the National Research Ethics Committee and the University of Manchester Research Ethics Committee. Patients and volunteers gave informed, written consent for their tissue samples, including plucked hair, to be used for this study. Scalp follicles were obtained following hair transplant procedures, and human skin was obtained following abdominal surgery. Scalp HSs were obtained from healthy volunteers by placing eyelash tweezers either side of a single HS, at the skin surface, and plucking forcefully. This resulted in anagen scalp hairs that retained part of the pigmented follicular matrix and ORS, as determined by light microscopy (Fig. 1a). Plucked hairs with a club hair were occasionally observed but were not used in the study.

### Cell culture

Human DP were isolated from anagen scalp follicles, as described [24]. DP were transferred into a 3.5 cm tissue culture plate in 2 mL Dulbecco’s modified Eagle’s medium (DMEM) with GlutaMAX (Life Technologies), 20% v/v foetal bovine serum (FBS; Life Technologies), penicillin (100 IU/mL), streptomycin (100 μg/mL), amphotericin B (2.5 μg/mL) and cultured at 37 °C/5% CO_2_. DP fibroblasts were sub-cultured at approx. 80% confluence using trypsin (0.05%)/EDTA (0.02%) in PBS (passage 1). After 24 h, medium was replaced with DMEM containing GlutaMAX, 10% FBS, without antibiotics or antimycotics.

DPS were created using the “hanging drop” method, as described [12], and cultured for 48 h at 37 °C/5% CO_2_ prior to analysis. To ensure consistency, only DPS that had formed a single, smooth spheroid in each hanging drop were used in experiments. Human dermal fibroblasts (DFs) were cultured from abdominal skin explants in 1 mL DMEM (supplemented as above)/20% FBS, and sub-cultured following 3 weeks of outgrowth, prior to expansion in supplemented DMEM/10% FBS. Cells were not used beyond passage 3. Cell viability in spheroids was assessed as previously described [12] by seeding spheroids onto tissue culture plastic in supplemented DMEM/10% FBS and observing cell outgrowth within 48 h.

### Plucked HS-DPS co-culture (HS-DPS)

**Fig. 2:**
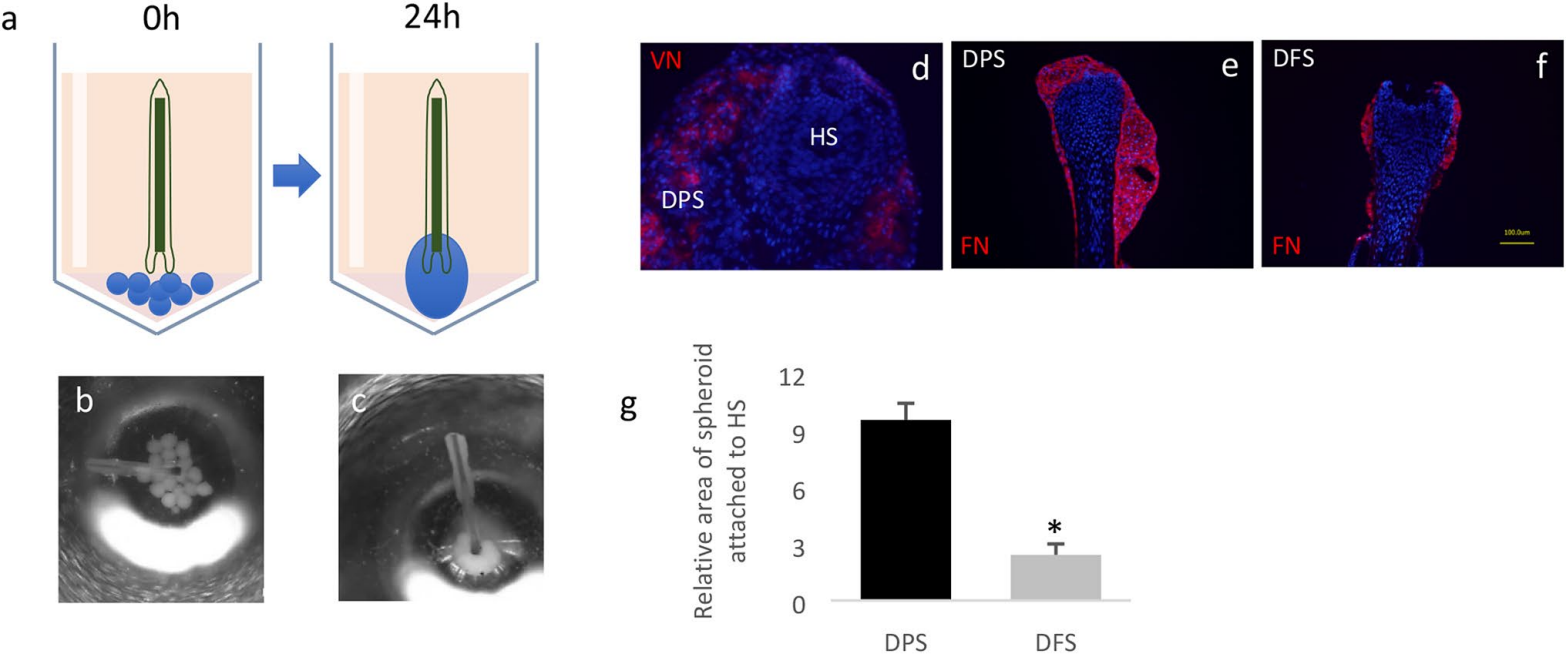
Schematic diagram showing set-up of HS-DPS model: a plucked HS (brown) is co-cultured with multiple DPS (blue circles) in a V-bottom 96 well. Because the surface of the well is not permissive, DPS adhere to each other and the HS within 24 h of culture (**a**). Macroscopic images of co-cultured HS-DPS at 0 h (**b**) and 24 h (**c**) of co-culture. HS-DPS at 24 h of co-culture showing anti-versican (VN) IR (red) localisation in the DPS (**d**). HS-DPS at 24 h of co-culture showing DPS [anti-fibronectin (FN) IR, red] attaching to and surrounding the plucked HS (**e**). Plucked HS cultured with spheroids containing human dermal fibroblasts (DFS) show significantly less attachment to the plucked HS (**f**, **g**; students *t* test; *p < 0.01, graph represents dermal papilla cells and dermal fibroblasts from three independent donors; error bars = standard error of the mean)

Twenty DPS were placed into a single well of a 96-well plate (Merck) containing 100μL DMEM (supplemented as described above; with 10% FBS). To facilitate DPS attachment to the plucked HS and prevent cell outgrowth, wells had a V-shaped bottom pre-coated with polyhydroxyethylmethacrylate (poly-HEMA; Merck; 20 mg/mL in 95% v/v ethanol). The hair fibre from freshly plucked scalp HS was dissected from the hair shaft and the presence of proximal follicle epithelium was assessed by light microscopy [4]. A single plucked anagen HS was placed into the centre of the well and oriented so that the proximal HS epithelium was in contact with the DPS (Fig. 2a, b). HS–DPS were incubated at 37 °C/5% CO_2_ for 24 h prior to analysis.

### Immunofluorescence microscopy

Tissue cryosections (7 μm) were fixed and processed as described in the table (Online resource 1). Cryosections were counterstained with 4′,6-diamidino-2-phenylindole (DAPI; 1 μg/mL) and mounted in Faramount (Dako). Terminal deoxynucleotidyl transferase dUTP nick end labelling (TUNEL) was carried out according to Manufacturer’s instructions (ApopTag^®^ apoptosis detection kit, Merck).

### Alkaline phosphatase activity

Cryosections were fixed in acetone at − 20 °C followed by two consecutive washes in phosphate-buffered saline (PBS). Alkaline phosphatase (ALP) activity was assessed using an ALP substrate assay kit (VECTOR blue), according to manufacturer’s instructions. The ALP inhibitor levamisole [8] was included as a negative control, according to manufacturer’s instructions (VECTOR).

### Quantitative immunohistomorphometry

Images were obtained by fluorescence microscopy (Keyence BZ-8000, Osaka, Japan) and quantified using ImageJ software (NIH, Bethesda, Maryland). The immunofluorescence intensity for K85 was compared by quantitative immunohistochemistry as previously described [10] and cell counts (Ki-67/TUNEL) were measured within a defined reference area that was consistent across all samples, as described (Online resource 2).

### Statistical analysis

Parametric data were assessed using the Student’s *t* test, and non-parametric data were assessed using the Mann–Whitney *U* test (GraphPad Prism, version 8). A value of *p* < 0.05 was considered significant.

## Results

Freshly plucked human anagen scalp HSs retained part of the proximal portion of the hair matrix and the inner root sheath as demonstrated histologically and by the presence of Ki-67 + proliferative matrix keratinocytes (Fig. 1a, b). Keratin-85 (K85) immunoreactivity (IR) was clearly localised to the pre-cortical matrix and proximal HS (Fig. 1c). No residual mesenchyme was present in plucked HS, as demonstrated by negative staining for anti-versican (VN) IR (Fig. 1d, e). As expected, DPS cultured for 48 h aggregated into single spheroids that expressed markers of DP inductivity, such as ALP activity [15], versican [26] and noggin [27] IR (Fig. 1f–h). Interestingly, the distribution of versican within cultured DPS was very heterogeneous (Fig. 1g). DP fibroblasts migrated out of DPS within 48 h of being re-seeded onto tissue culture plastic, demonstrating viability of the cells in 3D (Fig. 1i).

Within 24 h of co-culture, individual DPS coalesced and surrounded the remnant matrix of the plucked HS. At 24 h, HSs could be removed from culture along with the attached DPS, indicating that DPS had successfully formed a tissue unit with the matrix (Fig. 2a–c). Robust attachment between DPS and plucked HS occurred in 100% of co-cultures. Versican (VN) showed heterogeneous distribution in the attached DPS after 24 h of co-culture (Fig. 2d), as was observed in single DPS prior to co-culture (Fig. 1g), making it difficult to define the junction between DPS and plucked HS. The attached DPS was more easily distinguished from the plucked HS by the expression of fibronectin (FN, Fig. 2e), allowing accurate quantification of Ki67 + and TUNEL + cells in the HS epithelium alone. Interestingly, the ability of DPS to attach to plucked HSs in vitro after 24 h of culture was not observed with spheroids cultured from human dermal fibroblasts (DFS), as attachment to HS by DFS was significantly diminished compared to DPS (*p* < 0.01, Fig. 2e–g).

**Fig. 3:**
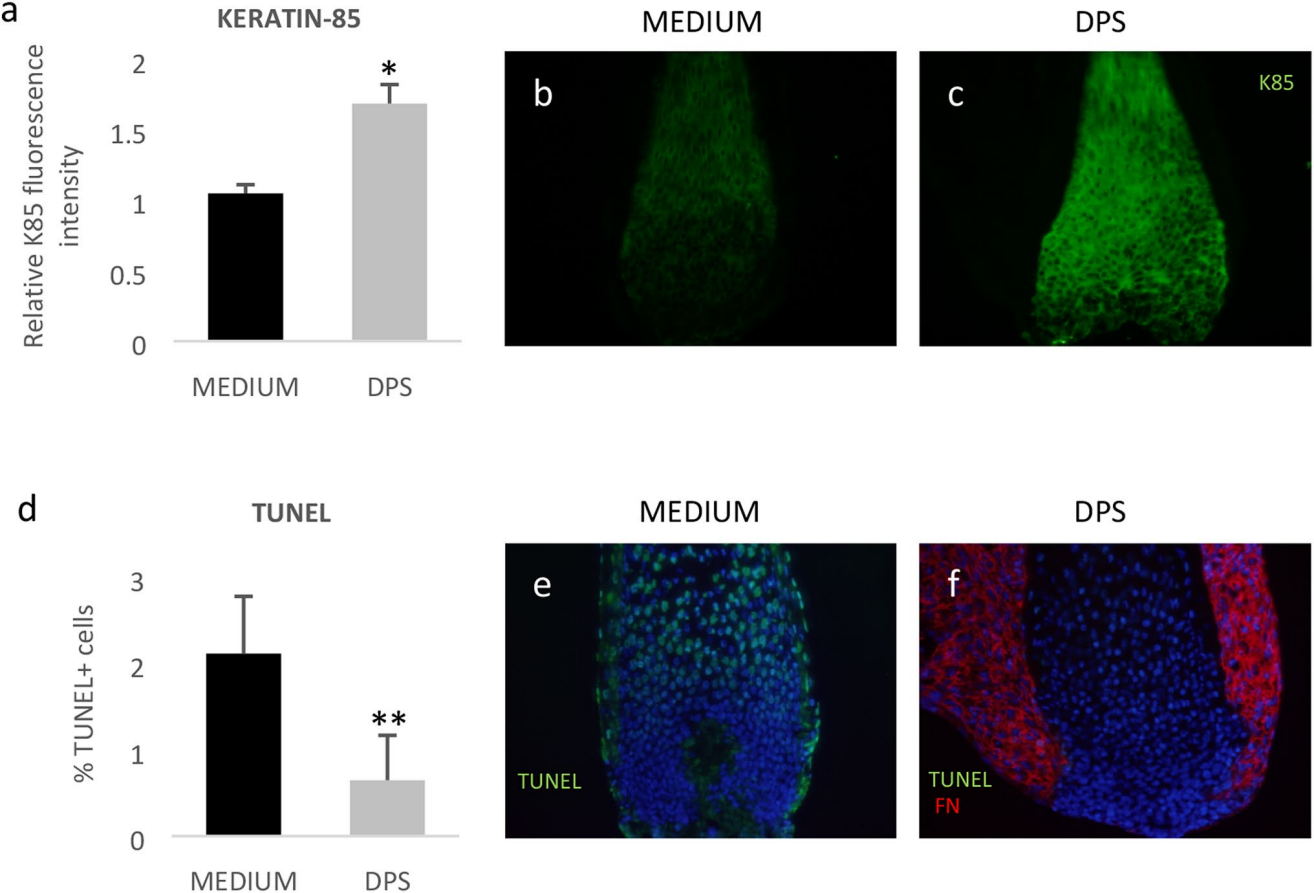
Anti-K85 IR is significantly increased in plucked HS after 24 h co-culture with attached DPS (**a**, **c**), compared to HS cultured in medium alone (**a**, **b**) (*p < 0.0001; error bars = standard error of the mean; medium group = 16 individual cultures; DPS group = 20 individual cultures. Plucked hair from 8 individual donors). Percentage of TUNEL + cells is borderline significantly decreased in plucked HS after 24 h co-culture with attached DPS (**d**, **f**), compared to HS cultured in medium alone (**d**, **e**) (**p = 0.0508; error bars = standard error of the mean; medium group = 9 individual cultures; DPS group = 10 individual cultures. Plucked hair from 5 individual donors). Red = anti-FN IR

Importantly, the presence of attached DPS significantly increased the intensity of K85 IR (*p* < 0.0001; Fig. 3a–c), and borderline significantly decreased the percentage of TUNEL + cells (*p* = 0.0508; Fig. 3d–f), in the proximal epithelium of the plucked anagen HS after 24 h culture, compared to plucked HSs cultured in medium alone. However, attached DPS did not prevent the rapid and complete loss of Ki67 + cells in the HS matrix (data not shown).

## Discussion

The pragmatic and physiologically relevant HF model we report here offers an instructive approach for investigating HF differentiation in the human anagen hair matrix. The experiments were designed primarily as a proof-of-principle study for the benefit of colleagues working in the field. It is, therefore, expected that future additional parameters will be assessed in this system, such as Wnt activity in the hair matrix, the gene expression profile of hair matrix keratinocytes and the impact of different culture media on supporting DP–hair matrix interactions.

This model contains the minimal cellular machinery for sustaining some aspects of epithelial–mesenchymal interactions that characterize human scalp HFs ex vivo. The increase in K85 in HS pre-cortical matrix cells with attached DPS attests to the vitality of pre-cortical human hair matrix keratinocytes, which are extremely difficult to culture, but which are shown here to continue synthesizing this major hair keratin [10, 17, 22]. The decrease in apoptosis in HF matrix cells with attached DPS suggests that some secreted components (“papilla morphogens”) [20] suppress hair matrix keratinocyte apoptosis under assay conditions; however, attached DPS did not prevent the complete loss of Ki67 + cells in the HS matrix, indicating that the secretory activity of DPS and the chosen assay conditions does not sufficiently support the extremely high proliferative activity of matrix keratinocytes. Thus, this co-culture system is, to the best of our knowledge, the first one to experimentally monitor human DP–hair matrix keratinocyte interactions in vitro.

Of interest is the heterogenous distribution of versican in DPS, which has previously been observed in human DP spheroid culture [11], and which suggests the presence of distinct cellular subpopulations in the human DP, as in murine DPs [28]. The same pattern of versican expression observed between single DPS and co-cultured DPS suggests that co-culture does not influence the distribution of versican in aggregated DP cells. DPS show significantly higher adhesion to the surface of the plucked HS compared to DFS, suggesting that DPS have an intrinsic affinity for the HS and can re-establish a unique epithelial–mesenchymal interface [18]. This is consistent with previous reports demonstrating that DP fibroblasts and fibroblasts from interfollicular dermis display key differences in their adhesion to basement membrane proteins, such as collagen-IV [2], and distinct expression of ECM components and ECM adhesion receptors [6, 25]. Differential adhesion of dermal fibroblast populations to the HF may also play a role in supporting HS survival, as human hair follicle survival in vitro is enhanced by activation of surface integrins [14]. However, critical components of DP-hair matrix interaction which sustain proliferation, such as P-cadherin expression, in the most proximal hair matrix cells at the DP interface [23] are clearly lacking in this system and may not easily be replaced.

In conclusion, this study describes the set-up of an organotypic HF model that provides the sophistication of the HF organ culture model with the flexibility of simpler in vitro organotypic HF models. As a tool for basic research, this pragmatic model provides a workable platform for investigating how exactly the DP controls cell proliferation and the differentiation of epithelial lineages in human hair.

## Supplementary Information

Below is the link to the electronic supplementary material.

Supplementary file1 (PDF 110 KB)

Supplementary file2 (PDF 126 KB)
