# Supplementary material for: Towards developing an organotypic model for the preclinical study and manipulation of human hair matrix-dermal papilla interactions
Source: Arch Dermatol Res. 2021 Jan 12;314(5):491–7. doi: 10.1007/s00403-020-02178-8 (PMC9163005; doi:10.1007/s00403-020-02178-8)
Supplement: Supplementary file 1 — Supplementary file1 (PDF 110 KB) [file 403_2020_2178_MOESM1_ESM.pdf]

Online Resource 1

Title: Towards developing an organotypic model for the preclinical study and manipulation of human hair matrix-dermal papilla interactions

Journal: Archives of Dermatological Research

Authors: Christopher I Platt*, Jeremy Chéret, Ralf Paus

Corresponding author: Christopher.platt@manchester.ac.uk. Division of Cell Matrix Biology and Regenerative Medicine, The University of Manchester, Manchester, UK

Antibodies, fixatives and blocking reagents used for immunofluorescence

Target	Protocol	Primary antibody	Secondary antibody
Versican	4% paraformaldehyde, 0.1% triton-X100, 3% BSA	Mouse anti-versican (1:400; Developmental Studies Hybridoma Bank, the University of Iowa)	Goat anti-mouse Alexafluor 488 and 594 (1:200; Life Technologies)
Ki-67	Acetone 10% normal goat serum	Rabbit anti-Ki-67 (1:100; abcam)	Goat anti-rabbit Alexafluor 594 (1:200; Life Technologies)
K85	Acetone 10% normal goat serum	Guinea pig anti-K85 antibody (1:1000; gift from Lutz Langbein)	Goat anti-guinea pig Alexafluor 488 (1:200; Life Technologies)
Noggin	Methanol, 10% normal goat serum	Rabbit anti-noggin (1:500; abcam)	Goat anti-rabbit Alexafluor 488 (1:800; Life Technologies)
Fibronectin	1% paraformaldehyde, ethanol: glacial acetic acid (1:1) 10% normal goat serum	Mouse anti-fibronectin antibody (1:1000; abcam)	Goat anti-mouse Alexafluor 594 (1:200; Life Technologies)
