# Supplementary material for: Towards developing an organotypic model for the preclinical study and manipulation of human hair matrix-dermal papilla interactions
Source: Arch Dermatol Res. 2021 Jan 12;314(5):491–7. doi: 10.1007/s00403-020-02178-8 (PMC9163005; doi:10.1007/s00403-020-02178-8)

Online Resource 2

Title: Towards developing an organotypic model for the preclinical study and manipulation of human hair matrix-dermal papilla interactions

Journal: Archives of Dermatological Research

Authors: Christopher I Platt*, Jeremy Chéret, Ralf Paus

Corresponding author: Christopher.platt@manchester.ac.uk. Division of Cell Matrix Biology and Regenerative Medicine, The University of Manchester, Manchester, UK

Image analysis and quantification

Keratin-85

Anti-K85 immunoreactivity (IR) in plucked HS was quantified by drawing a line around K85+ areas within the HS and quantifying staining intensity within the marked area using ImageJ software (NIH, Bethesda, Maryland)(a)

Quantification of TUNEL+ nuclei in the HS

The number of TUNEL+ nuclei was quantified in a rectangular reference area, superimposed onto the HS precortical matrix (b,c) by ImageJ. To minimise bias in the placement of the reference area (and subsequent cell counts), the reference area was placed at the most proximal region of the tissue section. In addition, in all images, manual placement of the reference area was carried-out only when the blue channel was selected, highlighting only DAPI+ cells (b). Only after placement of the reference area was the green channel selected, which highlighted TUNEL+ nuclei (c). The same size reference area was used for all images to quantify cell number (white triangles identify TUNEL+ cells)

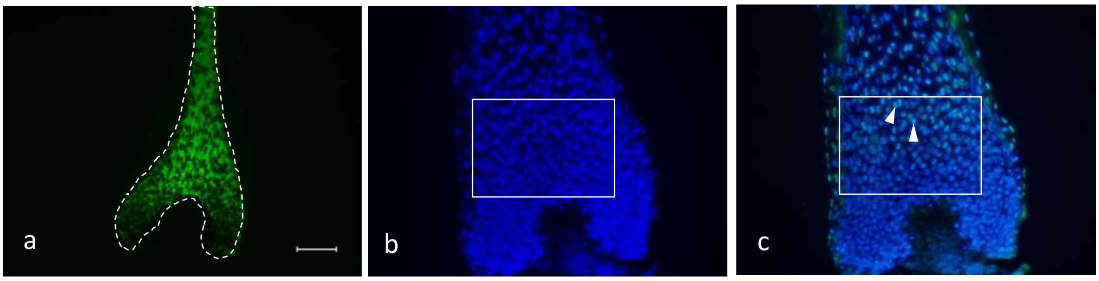

Supplement: Supplementary file 2 — Supplementary file2 (PDF 126 KB) [file 403_2020_2178_MOESM2_ESM.pdf]
